# Extracorporeal photopheresis as induction therapy in lung transplantation for cystic fibrosis: a pilot randomized trial

**DOI:** 10.3389/fimmu.2025.1583460

**Published:** 2025-05-16

**Authors:** Ilaria Righi, Claudio Fenizia, Daria Trabattoni, Mario Nosotti, Giacomo Grisorio, Claudia Vanetti, Sonia di Tella, Cristina Mocellin, Norma Fantini, Daniele Prati, Letizia Corinna Morlacchi, Valeria Rossetti, Francesco Blasi, Mario Clerici, Lorenzo Paolo Rosso

**Affiliations:** ^1^ Thoracic Surgery and Lung Transplant Unit, Fondazione Istituto di Ricovero e Cura a Carattere Scientifico (IRCCS) Ca’ Granda Ospedale Maggiore Policlinico, Milan, Italy; ^2^ Department of Pathophysiology and Transplantation, University of Milan, Milan, Italy; ^3^ Department of Biomedical and Clinical Sciences, University of Milan, Milan, Italy; ^4^ University of Milan, Milan, Italy; ^5^ Department of Psychology, Catholic University of the Sacred Heart, Milan, Italy; ^6^ Department of Transfusion Medicine and Hematology, Fondazione IRCCS Ca’ Granda Ospedale Maggiore Policlinico, Milan, Italy; ^7^ Respiratory Unit and Cystic Fibrosis Adult Center, Fondazione Istituto di Ricovero e Cura a Carattere Scientifico (IRCCS) Ca’ Granda Ospedale Maggiore Policlinico, Milan, Italy; ^8^ IRCCS Don C. Gnocchi Foundation, Milan, Italy

**Keywords:** lung transplanation, cystic fibrosis, photopheresis, immunomodulation, induction therapy

## Abstract

**Introduction:**

Extracorporeal photopheresis (ECP) is a viable treatment that slows the progression of chronic lung allograft dysfunction. Despite its immunoregulatory potential, data on extracorporeal photopheresis as an induction therapy remain rather limited.

**Methods:**

We conducted a pilot randomized controlled study on ECP as induction therapy in cystic fibrosis patients undergoing primary lung transplantation. Primary endpoints included safety, assessed based on the incidence of adverse events, treatment-related toxicity, and procedure-related complication rates; and feasibility, evaluated through the completion rate of scheduled ECP sessions, patient tolerability, and treatment discontinuation rates. Secondary endpoint consisted of an exploratory assessment of efficacy, using a composite measure that included three key components: freedom from biopsy-proven acute rejection within the first 12 months, absence of chronic lung allograft dysfunction at 36 months, and optimal graft function, defined as a predicted forced expiratory volume in the first second ≥ 90% at 36 months. Finally, exploratory endpoints included cell phenotypic and functional analyses, secreted immune protein profiling, and gene expression analysis for mechanistic insights. Patients were randomly assigned to receive either standard immunosuppressive therapy alone or standard therapy plus six sessions of extracorporeal photopheresis, with a follow-up period of 36 months.

**Results:**

Among 36 cystic fibrosis patients who underwent lung transplantation between 2018 and 2021 and met the eligibility criteria, 21 were randomized (9 to the study group and 12 to the control group). No patients in the treatment group experienced adverse events. The enrollment rate was 61%, and the treatment discontinuation rate was 22%. The clinical composite endpoint was achieved by 28.6% of patients in the treatment group and 16.7% in the control group. Exploratory endpoint analyses revealed significant decreases in pro-inflammatory cytokines, degranulating CD8^+^ T lymphocytes, and NK cells in the treatment group. Moreover, significant increases in Treg lymphocytes, IL-10-producing NK cells, and anti-inflammatory cytokines appeared to be associated with improved pulmonary function in the treatment group.

**Conclusions:**

Induction therapy with extracorporeal photopheresis is safe and feasible in lung transplantation for cystic fibrosis. Some clinical benefits appear to persist for the first 36 months of follow-up. Interestingly, a correlation between immunological modulation induced by extracorporeal photopheresis and pulmonary function was observed.

**Clinical Trial Registration:**

https://clinicaltrials.gov/study/NCT03500575?cond=NCT03500575&rank=1, identifier NCT03500575.

## 1 Introduction

Lung transplantation (LuTx) is the treatment of choice for several end-stage lung diseases, including cystic fibrosis (CF) but, despite improvements in surgical technique and the development of novel immunosuppressive and anti-infectious regimens, long time outcome after LuTx is not as good as in other solid organ transplantations (1). One of the reasons is that lung remains in continuous contact with the external environment: contact with pathogens can modify the delicate balance at the base of immune tolerance (2). Thus, the main factor limiting LuTx success is still the immune-mediated rejection (3).

Acute rejection (AR) is common in the first year after LuTx reaching an incidence up to 70% (4). Repeated episodes of AR, as well as lymphocytic bronchiolitis or diffuse alveolar damage, can trigger chronic lung allograft disfunction (CLAD) (5). The diagnosis of CLAD is extremely severe as CLAD is the leading causes of late morbidity and mortality considering that no effective therapies are available (6). Extracorporeal photopheresis (ECP) was proposed in the late ‘90s as a recommended adjunctive treatment to manage rejection in solid organ transplantation, including heart, lung and kidney (7). In retrospective analyses, ECP is associated with a relevant improvement in the clinical response to CLAD (8). A randomized clinical trial is now recruiting in UK to establish the improvement in survival of ECP added to standard care in recipients with CLAD (9).

Although the exact mechanism remains to be defined, ECP is believed to induce immune modulation, and in particular to upregulate T regulatory lymphocytes numbers and functions (10). More recently, ECP was suggested to modulate the activity of antigen presenting cells (APCs), namely dendritic cells, monocytes, and macrophages (11). The role of ECP as an induction therapy was first proposed by the Vienna group, who conducted an observational study involving 18 lung transplant recipients with chronic obstructive lung disease (COPD). In this study, nine patients received standard triple immunosuppressive therapy, while ECP was added to the regimen in the remaining nine patients. The results were encouraging, showing a reduction in episodes of higher-grade lymphocytic bronchiolitis and a significantly lower incidence of CLAD in the ECP group (12).

In February 2025, the European Respiratory Journal published the prospective, randomized, controlled trial conducted by the same research group between 2018 and 2020 to evaluate the efficacy of ECP as an induction therapy. Sixty-two recipients with COPD were randomized in a 1:1 ratio to receive either standard immunosuppressive therapy or ECP in addition to standard therapy. In the control group, 14 patients experienced one episode of acute cellular rejection (ACR), and one patient experienced three episodes; in the study group, only two patients experienced one episode of ACR. The authors concluded that the use of ECP as an induction therapy significantly reduced the incidence of acute rejection episodes and CLAD during the first 24 months following lung transplantation (13).

Based on this evidence, we designed a pilot randomized controlled trial to test the use of ECP immediately after LuTx in terms of safety, feasibility, preliminary efficacy, and mechanistic insights.

## 2 Materials and methods

### 2.1 Study design

This pilot study was a randomized clinical trial conducted by a middle-size lung transplant center. The study had a blinded outcome assessment; specifically, pulmonologists and biologists responsible for clinical and laboratory evaluations were blinded to treatment allocation, while patients were aware of their assigned intervention. The study protocol was registered on Clinicaltrials.gov (NCT03500575) and approved by the Hospital Institutional Review Board (ref. 1431/2017). We selected cystic fibrosis patients due to their high rejection risk and inflammatory burden. Additionally, they are typically young and resilient, and their lifelong disease management fosters strong engagement in care, enhancing study adherence and follow-up rates. Adult patients (≥18 years) with end stage cystic fibrosis requiring a double lung allograft were eligible for the study. Patients signed a written consent at the listing and were free to withdraw from the study at any time-point. Exclusion criteria were body weight < 40 Kg, re-transplantation, previous transplantation of other organs, multiorgan transplantation, and listing in the emergency program (13). A computed randomization was planned for patients who returned to the ward from the intensive care unit in less than 72 hours.

**Figure 1:**
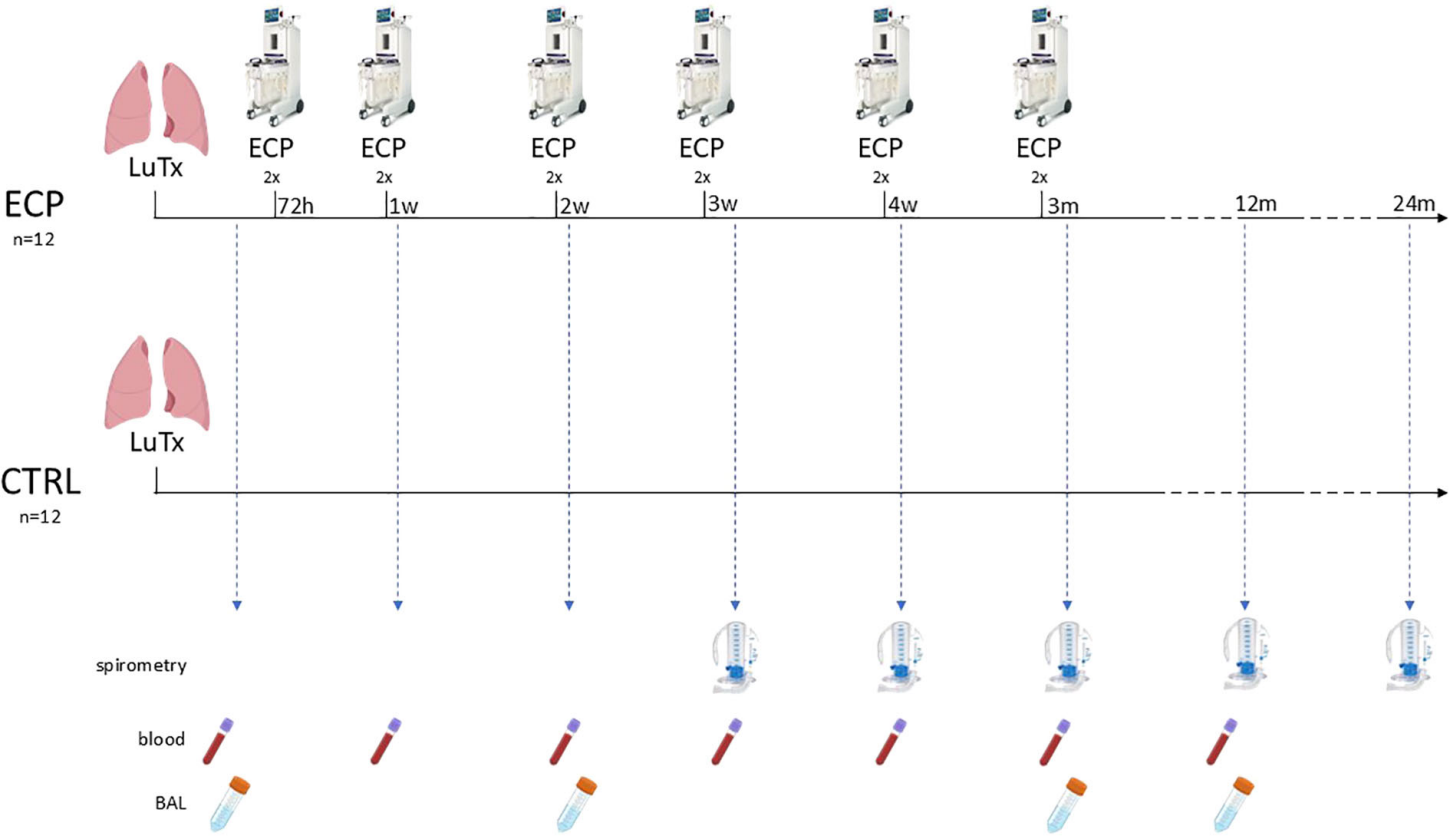
Graphical representation of the treatment protocol and patient surveillance. Analyses included routine laboratory tests, lung function assessment via spirometry at least three weeks post-transplantation, and documentation of adverse events. Surveillance bronchoscopy for infection screening was performed at weeks 1, 2, and 3, while transbronchial biopsies for rejection monitoring were conducted at weeks 12, 24, and 54. For study purposes, clinical follow-up continued for up to 36 months. ECP, extracorporeal photopheresis group; CTRL, control group; LuTx, lung transplantation.

**Figure 2:**
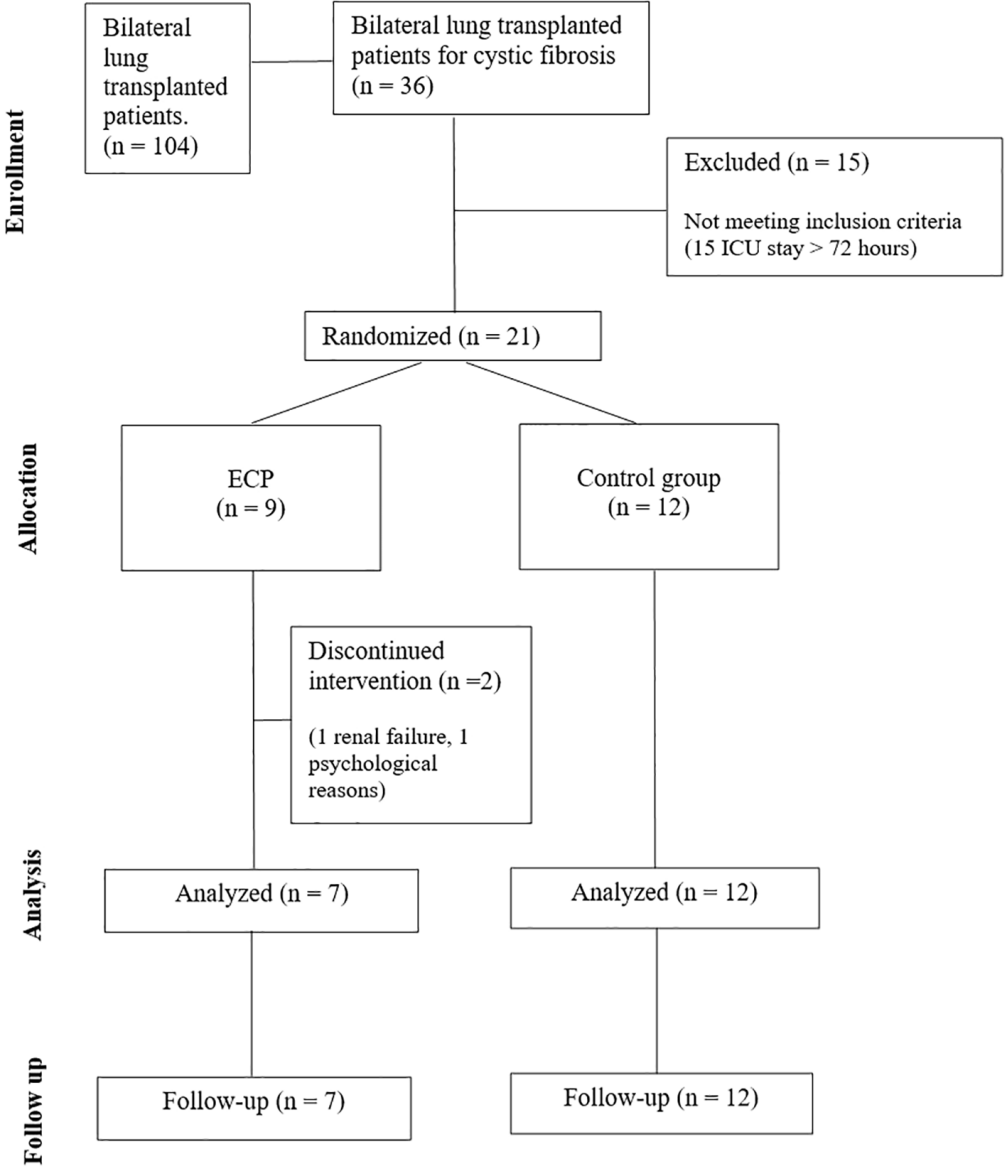
CONSORT flow diagram displaying the patient inclusion process.

All subjects received immunosuppressive therapy and anti-microbial prophylaxis according to local clinical practice (See **Supplementary 1**). In addition, patients in the interventional group (ECP group) underwent six sessions of ECP, each session consists in two treatments in consecutive days. The first session started 72 hours after LuTx and was followed by a weekly session 3 times, and 2 more sessions monthly in the following 2 months. In total, six sessions corresponding to 12 treatments were scheduled for each patient. For treatment protocol see **Supplementary 2**. Three years follow-up was planned as follows: a weekly visit for the first two months, then a visit every two weeks until the sixth month, then a monthly visit until the end of the study. Analyses included routine laboratory tests, monitoring of infections, lung function tests and registration of any adverse event. Bronchoscopy for surveillance of infections was performed at week 1, 2, and 3 after LuTx; transbronchial biopsies (TBB) with bronchoalveolar lavage (BAL) were performed at month 3, 6 and 1 year after LuTx or in case of new respiratory symptoms and/or a decrease greater than 10% in percent predicted forced expiratory volume in the first second (ppFEV1) (**Figure 1**). In case of diagnosis of AR, the patients were treated with pulsed corticosteroids (10mg/kg). Data was entered into an electronic case report form. The CONSORT Statement was followed (**Figure 2**).

### 2.2 Outcomes

The primary endpoints were safety and feasibility. Safety was based on the incidence of adverse events, treatment-related toxicity, and procedure-related complications. Feasibility was assessed through the completion rate of scheduled ECP sessions, patient tolerability, and treatment discontinuation rate.

The secondary endpoint consisted in the preliminary evaluation of efficacy. We designed a composite endpoint to capture the overall effectiveness of ECP in preventing rejection and preserving lung function. Our composite endpoint consisted of three key components: AR-free survival (no biopsy-proven acute rejection within the first 12 months), CLAD-free survival (no development of CLAD at 36 months), and optimal graft function (defined as ppFEV_1_ ≥ 90%). Acute cellular rejection was evaluated using histopathological interpretation of transbronchial biopsies based on ISHLT criteria (14). For the purpose of this study, AR was defined as a histological grade ≥ A1 rejection at transbronchial biopsy. Lymphocytic bronchiolitis as well as antibody mediated rejection were defined following international consensus (14, 15). CLAD was defined by a decrease in the ppFEV1 of at least 20% from the best baseline value in absence of other identifiable causes such as AR, infection or anastomotic problems (16, 17). Cytomegalovirus reactivation was defined as a quantitative CMV-DNA level >200 IU/ml.

As exploratory endpoints, we examined mechanistic insights, including cell phenotypic and functional analyses, secreted immune protein profiling, and gene expression analysis. The immunophenotypic subpopulations were identified by flow cytometry on PBMCs. The following antibodies were employed: CD3-FITC (Beckman Coulter, California, USA), CD4-PC7 (Beckman Coulter), FoxP3-APC (Invitrogen, Massachusetts, USA), RoRγt-PE (Invitrogen), Tbet-PE (Invitrogen), CD56-PC7 (Beckman Coulter), CD16-PC5 (Beckman Coulter), CD25-PC5 (Beckman Coulter), PD1-APC (Invitrogen), PD1L-PerCP (R&D systems, Minnesota, USA), IL10-PE (Biolegend, California, USA), IL17-FITC (Invitrogen), IL21-APC (Invitrogen), IFNγ-FITC (Biolegend), TNFα-PE (Biolegend), granzyme-PE (Invitrogen), perforin-APC (Invitrogen). Cells were then washed with phosphate buffered saline (PBS, Euroclone, Milan, Italy) and fixed with paraformaldehyde (PFA, Sigma-Aldrich) 1%. Cells were gated based on forward (FSC) and side scatter (SSC) properties, and lymphocytes were identified. Then, T helper lymphocytes were identified based on the CD3 and the CD4 staining, and further subpopulations based on FoxP3, RoRγt and Tbet gating. Natural Killer identification was based on the CD56 and CD16 staining. Finally, activation markers and/or cytokines expression were evaluated on cells; at least 100,000 events were acquired using a CytoFLEX S flow cytometer; data were analyzed using the Kaluza 2.0 software (Beckman Coulter).

### 2.3 Statistical analysis

The 1:1 randomization was done with “random allocation rule”. The study is single-blind regarding investigators who assess the outcomes. Researchers who assess the outcomes received biological samples and medical records masked for group allocation. We hypothesize that the immunoregulatory property of ECP could promote graft tolerance immediately after lung transplantation; this was formally translated in difference of incidence of AR between the ECP-treated group and the non-treated group.

Sample size calculation: considering *two*-*proportion z*-*test*, setting type I and type II statistical error as 0.25 and 0.20 respectively, and using 0.35 as acute rejection incidence in non-treated group (18) and 0.04 as expected acute rejection incidence in treated group, the sample size was 11 (+1 lost at FU) subjects for each group (total 24 subjects). The study used *per protocol* analysis.

For the study variables, medians and ranges were reported for quantitative variables. T-test and Linear model were considered significant when *p* value ≤0.05. Size effect was considered large when η^2^p≥0.14, intermediate when 0.14>η^2^p≥0.06. When appropriate, for size effect analysis, Cohen’s d ≥0.8 was considered large, while intermediate when 0.8>d≥0.5.

Before proceeding to the extraction of the components, suitability analyses were performed. First, we analyzed the total-item correlation for the initial assessment and refinement of the variables. Items displaying a low correlation (Oblimin >0.3) were removed from further analyses. Then, we employed the Kaiser–Meyer–Olkin measure of sampling adequacy (KMO) to verify sample adequacy requirements. In this case, values above 0.5 were considered acceptable (19) and taken into account for further analyses. The Bartlett’s Test of Sphericity should be significant (p <.05) for factor analysis to be suitable. Furthermore, before proceeding with the components, we inspected the correlation matrix looking out for very high correlations (r >0.9) to avoid extreme multicollinearity.

The analyses were performed using Jamovi Software (20, 21) and GraphPad Prism 8. Graphs and images were assembled by GraphPad Prism 8 and Biorender.com, respectively.

All the procedures were carried out in accordance with the GLP guidelines adopted in our laboratories.

## 3 Results

From September of 2018 until February 2021, a total of 36 patients were transplanted and assessed for eligibility, of whom 21 were randomized (**Figure 2**). Subsequently, the enrolment was discontinued due to the COVID-19 pandemic which resulted in a complete reorganisation of the hospital and a drastic reduction in surgical departments. Twenty-one patients were enrolled in the study, of whom 9 was randomly assigned to ECP group and 12 to control group (CTRL). None of the patients in the treatment group experienced adverse events, treatment-related toxicity, or procedure-related complications, indicating an optimal safety profile of the ECP. All scheduled ECP sessions were completed (100% completion rate); however, patient tolerability was 86%, as one patient withdrew due to psychological challenges. Overall, the treatment discontinuation rate was 22%, as we halted ECP in one patient due to persistent borderline renal function.

### 3.1 Clinical outcomes

The two groups were well-balanced at baseline clinical parameters and were homogeneous for lung allocation score and perioperative outcomes (**Table 1**). The number of clinically relevant infections was slightly higher among control group patients (N=4) compared to the ECP group (N=3). Additionally, serological CMV reactivation was observed in seven patients, all of whom belonged to the control group.

**Table 1:** Baseline characteristics.

	CTRL (control group)	ECP (treatment group)	*P* value (T-Test)
**Age**, mean (SD)	32.08 (11.2)	31.34 (4.96)	0.886
**Gender,** M/F n (%M)	5/7 (41.7)	3/6 (33.3)	0.568
**CMV mismatch,** n (%)	4 (33.3)	0 (0)	0.086
**ECMO,** n (%)	8 (66.7)	5 (71.4)	0.829
**LAS**, mean (SD)	40.86 (7.53)	42.25 (6.24)	0.687
**MV**, hours, mean (SD)	25.25 (15.57)	25.71 (10.24)	0.945
**ICU stay** (days), mean (SD)	3.33 (1.44)	2.43 (0.54)	0.131
**Discharge (**days), mean (SD)	23.42 (10.26)	24 (4.08)	0.888

CTRL, control group; ECP, treatment group (extracorporeal photopheresis); CMV, cytomegalovirus; ECMO, extracorporeal membrane oxygenation; LAS, lung allocation score; MV, mechanical ventilation time; ICU, intensive care unit.

**Figure 3:**
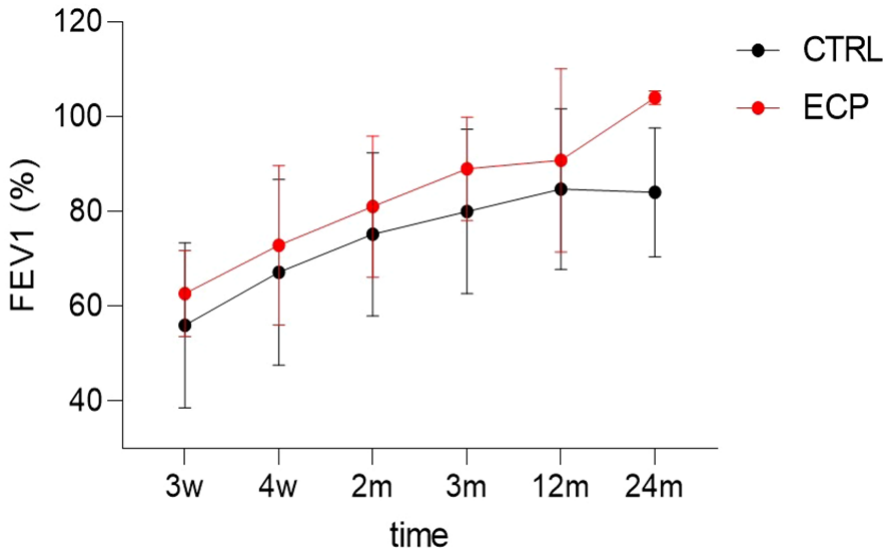
Diagram of the best FEV_1_ dynamics over 24 months. Time factor linear model p<0.001, size effect η^2^p=0.258. Group factor linear model p=0.035, size effect η^2^p=0.595. ECP, extracorporeal photopheresis group; CTRL, control group; FEV1, percent predicted forced expiratory volume in 1 second.

The number of patients with AR episodes was identical in both groups at the 12-month follow-up (n=3 per group). Moreover, at the 36-month follow-up, one patient in each group developed CLAD. Graft function, assessed by ppFEV1 measurements at the designated time points, was significantly better in ECP patients; this difference was mostly evident at the 24-month time point (linear model p <0.001, size effect η2p 0.258. group factor p value 0.035, size effect η2p 0.595) (**Figure 3**). A ppFEV1 greater than 90% was recorded in four patients in each group at the 36-month follow-up. The composite clinical endpoint was reached by 28.6% of patients in the ECP group, whereas 16.7% of those in the control group achieved it.

### 3.2 Cell phenotypic and functional analysis

**Figure 4:**
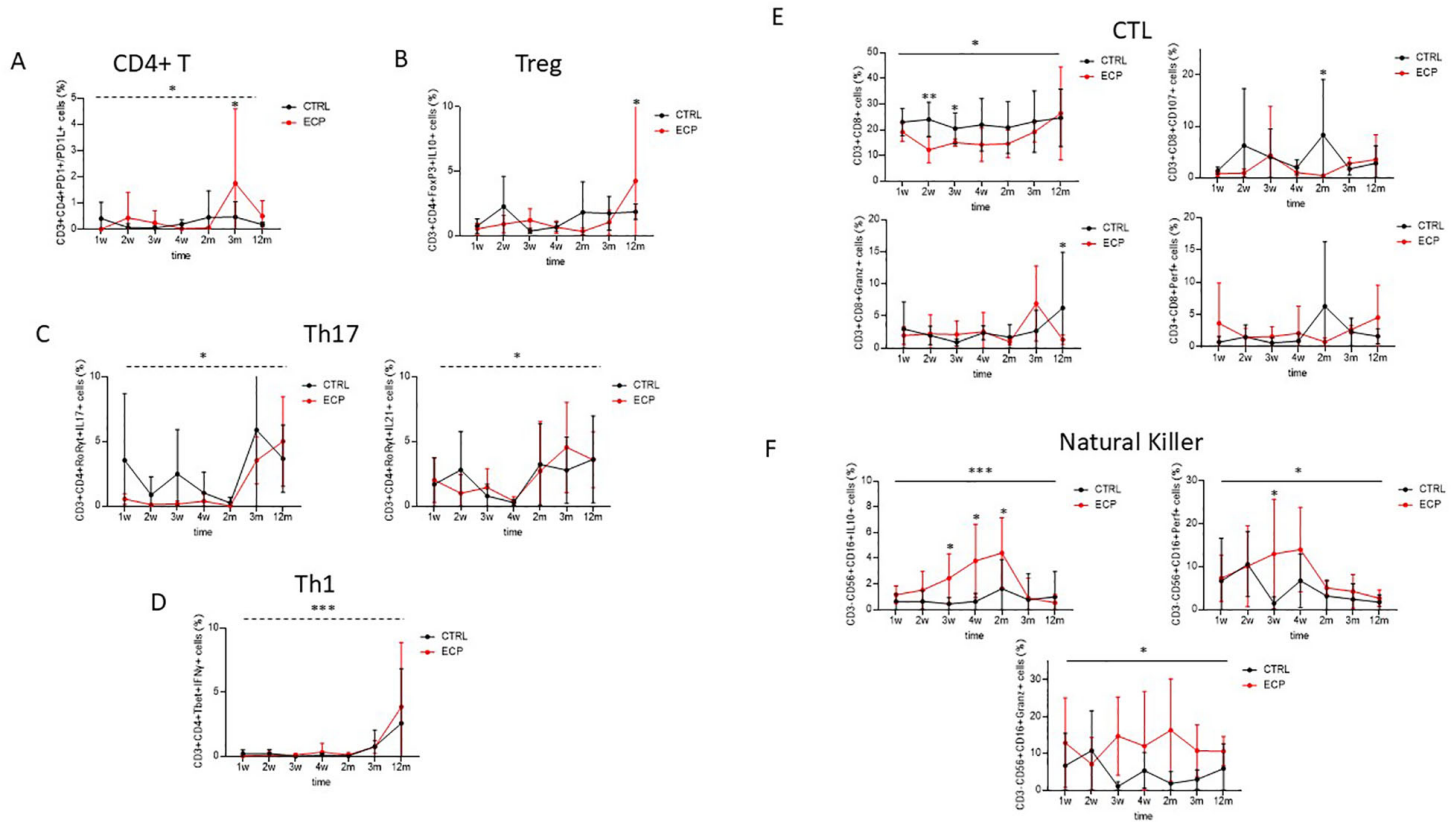
Cell phenotypic and functional analyses were assessed in control group (CTRL, in black) and ECP group (ECP, in red). **(A)** Double positive PD1+/PD1L+ expressing CD4+ T lymphocytes were evaluated. Time factor (dashed line) linear model p=0.032, size effect η^2^p=0.202. Pairwise comparison at month 3 was p=0.008. **(B)** T regulatory lymphocytes. IL10 expressing T regulatory lymphocytes (Treg: CD3+CD4+CD25+FoxP3+). Pairwise comparison at month 12 was p=0.05. **(C)** T helper 17 lymphocytes. Left panel: IL17 expressing T helper 17 lymphocytes (Th17:CD3+CD4+RORγt+). Time factor (dashed line) linear model p=0.014, size effect η^2^p=0.253. right panel: IL21 expressing Th17. Time factor (dashed line) linear model p=0.012, size effect η^2^p=0.262. **(D)** T helper 1 lymphocytes. IFNγ expressing T helper 1 lymphocytes (Th1: CD3+CD4+Tbet+). Time factor (dashed line) linear model p<0.001, size effect η^2^p=0.312. **(E)** Cytotoxic T cell. Upper left panel: cytotoxic T cell (CTL: CD3+CD8+). Group factor linear model p=0.025. Pairwise comparison at weeks 2 and 3 were p=0.002 and p=0.040, respectively. Upper right panel: Degranulating (CD107+). Group factor linear model p=0.057. Pairwise comparison at month 2 was p=0.038. Lower left panel: Perforin expressing (Perf+) CTL. Lower right panel: Granzyme expressing (Granz+) CTL. Pairwise comparison at month 12 was p=0.046. **(F)** Natural Killer. Upper left panel: IL10 expressing (IL10+) Natural Killer (NK: CD3-CD15+CD56+). Group factor linear model p<0.001, size effect η^2^p=0.163. Pairwise comparison at week 3, week 4, month 2 was p=0.030, p=0.003 and p=0.043, respectively. Upper right panel: Perforin expressing (Perf+) NK. Group factor linear model p=0.047, size effect η^2^p=0.061. Pairwise comparison at week 3 was p=0.015. Lower panel: Granzyme expressing (Granz+) NK. Group factor linear model p=0.012, size effect η^2^p=0.039. ***p<0.001, **p<0.01, *p<0.05.

The main subsets of T cell lymphocytes and NK cells were assessed by flow cytometry. Results showed that PD1- and PDL1-expressing T helper lymphocytes were significantly increased over time (time factor linear model p=0.032, size effect η^2^p=0.202) (**Figure 4A**). Such an increase was independent from treatment, although the pairwise comparison showed these cells to be significantly upregulated at month 3 in ECP compared to CTRL patients (*p*=0.008). Immunosuppressive T regulatory lymphocytes (Treg; defined as CD3+CD4+FoxP3+) expressing interleukin (IL)10 were significantly increased in ECP at month 12 (Pairwise comparison *p*=0.05) (**Figure 4B**). Pro-inflammatory T helper 17 lymphocytes (Th17; defined as CD3+CD4+RoRγt+) expressing either IL17 or IL21 increased overtime, regardless of the treatment (time factor linear model *p*=0.014, size effect η^2^p=0.253 and *p*=0.012, size effect η^2^p=0.262, respectively) (**Figure 4C**). T helper 1 (Th1; defined as CD3+CD4+Tbet+) increased at later timepoints as well, regardless of the treatment (time factor linear model p<0.001, size effect η^2^p=0.312) (**Figure 4D**).

Cytotoxic T lymphocytes (CTLs) were steadily decreased in ECP patients (group factor linear model *p*=0.025, size effect η^2^p=0.196), this was most evident at week 2 and 3 (pairwise comparison *p*=0.002 and *p*=0.040, respectively) (**Figure 4E**). In ECP compared to CTRL patients, degranulating CTLs (CD3+CD8+CD107+ cells) were significantly reduced at month 2 (pairwise comparison *p*=0.038); finally, granzyme positive CTLs were significantly downregulated in these patients at month 12 (pairwise comparison *p*=0.046).

Natural killer cells (NK; defined as CD3-CD56+CD16+ cells) expressing IL10 were steadily increased in ECP patients (group factor linear model *p*<0.001, size effect η^2^p=0.163. Pairwise comparison at week 3, week 4, month 2: p=0.030, *p*=0.003 and *p*=0.043, respectively) (**Figure 4F**). Similarly to what was observed in CD8+ T lymphocytes, both perforin and granzyme-containing NK cells were significantly reduced ECP patients alone (group factor linear model *p*=0.047, size effect η^2^p=0.061 and *p*=0.012, η^2^p=0.039, respectively).

### 3.3 Secreted immune proteins analysis

**Figure 5:**
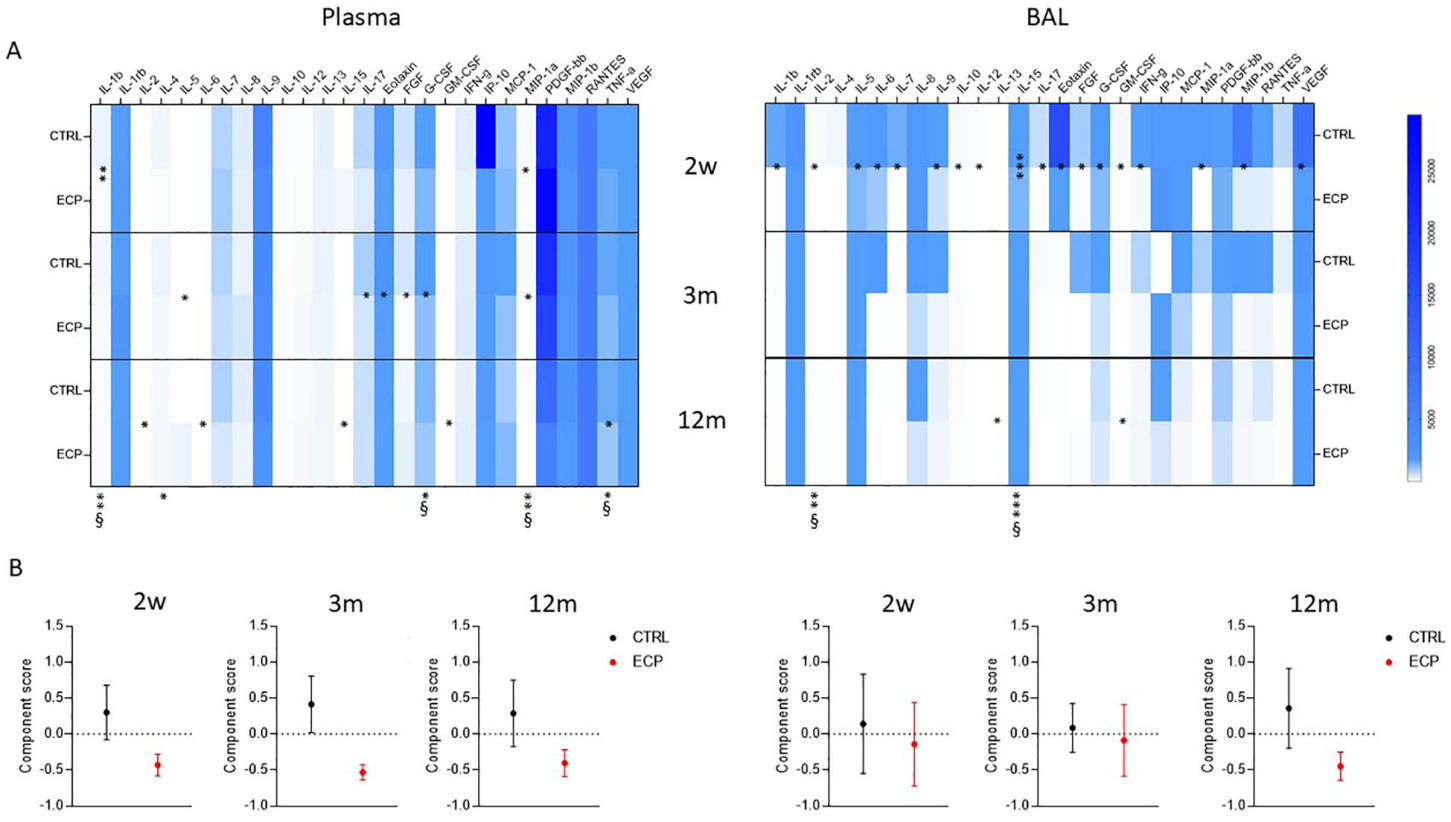
Secreted soluble factors. **(A)** Secretome analysis. Secreted soluble factors were evaluated (upper panels) in plasma (left) and BAL (right) control group (CTRL) and ECP group (ECP) at week 2, month 3 and 12. Values are expressed in pg/ml. Group factor linear model statistical significance scores are displayed below the heatmap, while the pairwise comparison on the corresponding slots (***p<0.001, **p<0.01, *p<0.05. § large effect size). **(B)** Component analysis (lower panel) scores performed on plasma (left) and BAL (right) soluble factors are displayed for control group (CTRL, in black) and ECP group (ECP, in red) at month 3 and 12. (Student’s t test p=0.057, size effect Cohen’s d=1.04).

Cytokines and chemokines concentration was analyzed in plasma and BAL samples of all patients at week 2, month 3 and 12 (**Figure 5A**). Overall results showed a down-regulation in the concentration of pro-inflammatory cytokines in BAL and, more robustly, in plasma of ECP compared to CTRL patients. Thus, in ECP compared to CTRL patients IL1β, IL4, G-CSF, MIP1α and TNFα production was significantly reduced throughout the study period (p<0.01 large effect size, p<0.05, p<0.05 large effect size, p<0.01 large effect size, p<0.05 large effect size, respectively) in plasma, whereas IL2 and IL15 were significantly reduced in BAL (p<0.01 large effect size, p<0.001 large effect size, respectively).

Principal component analysis identified a single component discriminating the two treatments (**Figure 5B**). The factors partaking such components are listed in **Supplementary Table 1**. In plasma, the component score showed a pronounced difference starting at week 2, which was maintained through month 3 (Student’s t test *p*=0.057, size effect Cohen’s d=1.04) and partially waned by month 12. Overall, the ECP displayed decreased values of the component score, indicating the presence of an anti-inflammatory milieu. In BAL, a different trend was observed: thus, similar scores were observed in CTRL and ECP patients at week 2 and month 3, which then diverged from each other overtime (month 12), with ECP patients displaying a lower component score, i.e. the presence of an anti-inflammatory milieu.

### 3.4 Gene expression analysis

**Figure 6:**
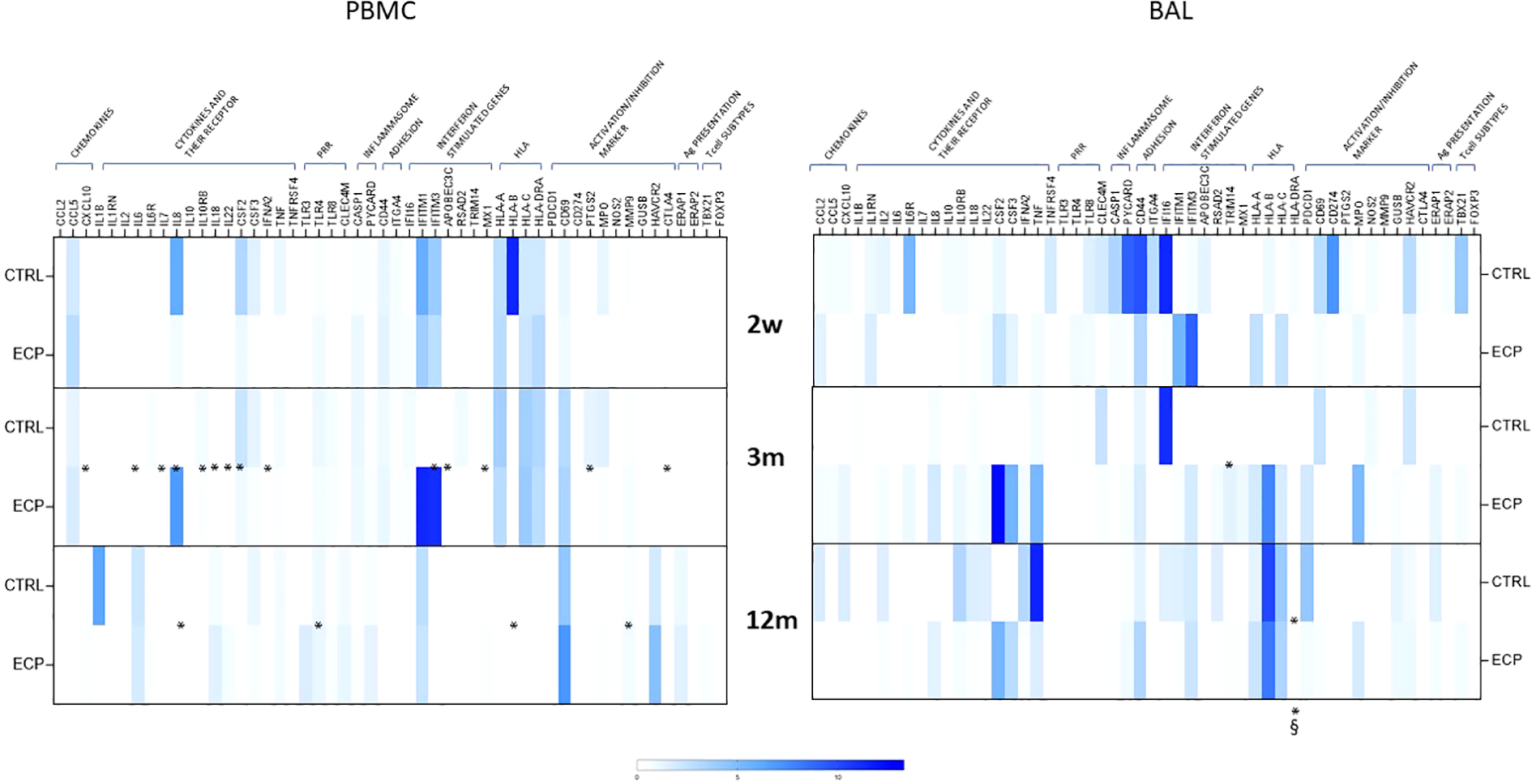
Gene expression analysis. mRNA expression of 84 genes was performed on PBMCs (left panel) and BAL (right panel) in control group (CTRL) and ECP group (ECP) at week 2, month 3 and 12. Values are expressed in fold induction over five housekeeping genes. Group factor linear model statistical significance scores are displayed below the heatmap, while the pairwise comparison on the corresponding slots (***p<0.001, **p<0.01, *p<0.05. § large effect size).

A 70-gene mRNA expression profiling was performed in PBMC as well as in cells isolated from BAL at week 2, month 3 and 12 (**Figure 6**). Genes were grouped in categories (chemokines, cytokines and cytokine receptors, pathogen recognition receptors (PRR), inflammasome, adhesion molecules, interferon stimulated genes (ISG), HLA, activation or inhibition markers, antigen presentation and transcription factors peculiar of T cell subtypes). ECP patients were characterized by a consistently reduced PBMC activation pattern at month 3. Notably, in BAL, class II HLA-DR expression was significantly lower in ECP across all timepoints (group factor linear model *p*<0.05, large effect size). Overall, a consistent signature related to ECP treatment could not be identified. Principal component analysis indicated that such items could not be factorizable.

### 3.5 Correlation analysis

**Figure 7:**
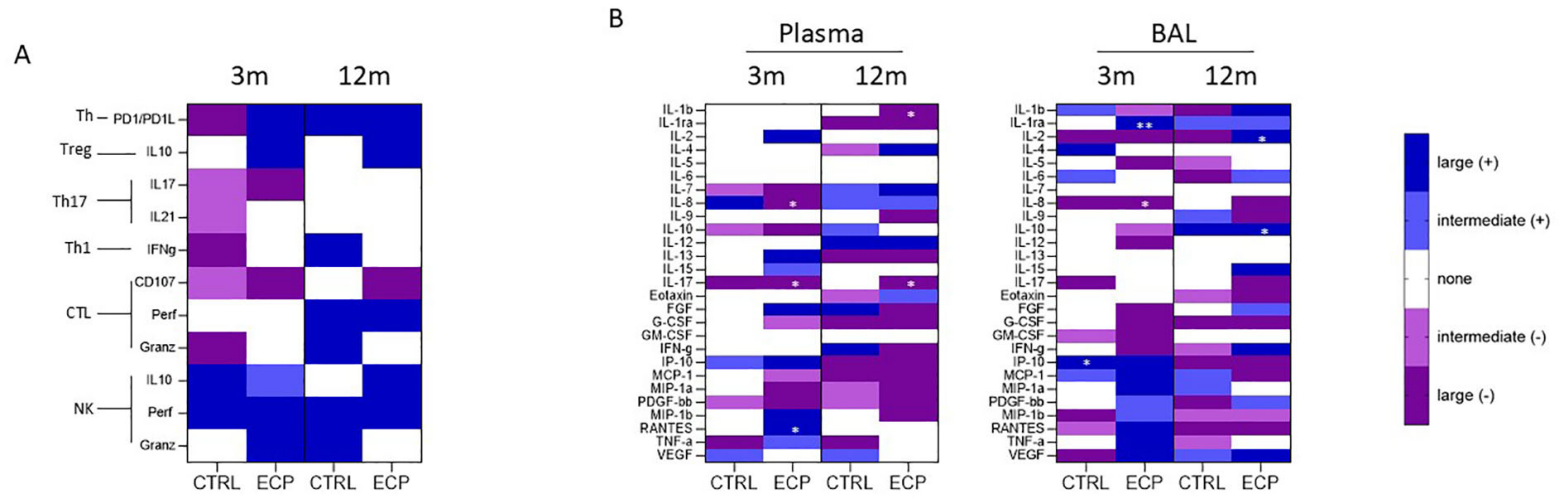
Correlation analysis: **(A)** FEV_1_/flow cytometric analysis correlation was performed at month 3 and 12 in CTRL and ECP groups. The color coding is relative to the effect size (where the dark blue/violet means large effect - Pearson’s r> ± 0.5, light blue/violet means intermediate effect - ± 0.5>Pearson’s r> ± 0,3, while white 0,3>r>-0,3). Moreover. blue is assigned to positive correlation (+), while violet to negative correlation (-). **(B)** FEV_1_/soluble factors analysis correlation was performed at month 3 and 12 in CTRL and ECP groups in plasma (left panel) and BAL (right panel). *p<0.05, **p<0.01.

To better identify which of the analyzed parameters was more likely to be clinically relevant, a correlation analysis between ppFEV_1_ and immunological parameters was performed at three and 12 months. Correlations between ppFEV_1_ and immune-phenotypical results are presented in **Figure 7A**; no statistical significance was reached for any of the parameters considered.

Analyses of possible correlations between ppFEV_1_ and the production of immune proteins, though, allowed the identification of distinct patterns in both plasma and BAL in ECP patients, (**Figure 7B**). Indeed, in ECP patients ppFEV_1_ was inversely correlated with pro-inflammatory cytokines including: 1) plasma and BAL IL8 (month 3, *p*<0.05, large effect size); 2) plasma IL17 both at month 3 and 12 (*p*<0.05, large effect size); and 3) plasma IL1β at month 12 (*p*<0.05, large effect size). Notably, direct correlations between ppFEV_1_ and anti-inflammatory proteins in BAL, including the decoy IL1 receptor IL1ra (month 3, *p*<0.01, large effect size) and IL10 (month 12, *p*<0.05, large effect size) were detected as well. Finally, ppFEV_1_ directly correlated with plasmatic IL2 as well (month 12, *p*<0.05, large effect size) in ECP patients.

## 4 Discussion

To the best of our knowledge, this pilot study is the second prospective, randomized, controlled trial evaluating ECP as induction therapy in lung transplantation. Unfortunately, the outbreak of the COVID-19 pandemic prevented us from reaching our planned sample size. Despite this setback, we took care of the safety and feasibility of ECP as an induction therapy in our surgical department. Communication of the study’s objectives and obtaining informed consent during patient listing proved quick and effective; no candidate declined participation, yielding a recruitment rate of 100%. However, the enrollment rate was 61%, primarily due to the stringent inclusion criteria emphasizing rapid ICU discharge and satisfactory renal function. The acceptance of the procedure was high (86%), with only one young patient discontinuing the therapy after two treatments because he felt overwhelmed. With the inclusion of a second patient who discontinued treatment after four sessions due to persistent borderline renal function, the overall discontinuation rate reached 22%. In contrast, a similar randomized trial investigating the use of ECP as prophylaxis for graft-versus-host disease in patients with hematologic malignancies reported a dropout rate of 43% (22). These findings suggest that lung transplant recipients with cystic fibrosis may be more inclined to tolerate the discomfort associated with the treatment compared to onco-hematological patients. The machine setup required that a dedicated doctor and nurse remain in the ward for three hours. Additionally, the “on-line” procedure necessitates venous access of an appropriate caliber, which in turn requires the expertise of properly trained nurses. These factors should be taken into account when assessing feasibility and conducting a cost analysis.

ECP as induction therapy demonstrated excellent safety, as no side effects directly attributable to the procedure were observed. Although the overall infection rates were similar between the two groups, it is noteworthy that 60% of patients in the control group experienced serological CMV reactivation, whereas none of the patients in the ECP group did. This discrepancy may be partly due to the high prevalence of CMV mismatch in the control group and the small sample size. Nevertheless, it is reassuring that no patients treated with ECP exhibited CMV reactivation.

To capture the overall effectiveness of ECP in preventing rejection and preserving lung function in our pilot study, we designed a composite endpoint encompassing a three-year follow-up period. The proportion of patients meeting each of the three endpoint components (12-month AR-free survival, 36-month CLAD-free survival, and 36-month ppFEV_1_ ≥ 90%) was consistently higher in the ECP group than in the control group. The functional outcome warrants additional commentary. ppFEV_1_ can be negatively influenced by various factors, including lung infection, pleural effusion, diaphragmatic dysfunction, chest wall stiffness or instability, size discrepancy, and limited rehabilitation potential (23). We meticulously evaluated each potential confounding factor that could have affected lung function. However, the ECP group still exhibited a significantly better ppFEV_1_ than the CTRL group, even several months post-surgery.

Our exploratory endpoints included cytokine testing, given the well-established correlation between cytokines and airway inflammation, as well as the relationship between anti-inflammatory cytokines and ppFEV_1_ (24, 25). Actually, we have shown the inverse relationship between pro-inflammatory cytokines and ppFEV1 in the ECP patients; this result supports the hypothesis that immunomodulation caused by ECP may allow a favorable milieu at the level of the small airways. The modification of immune responses induced by ECP was complex and multifaceted. ECP reduced the production of pro-inflammatory cytokines while increasing the levels of the anti-inflammatory cytokine IL-10 in both plasma and BAL. This modification was confirmed through gene expression analyses, which consistently showed downregulation of immune activation-related genes by ECP. Notably, HLA-D expression was also reduced, further supporting the role of ECP in down-modulating immune activation. Principal component analysis identified a distinct component that effectively differentiated LuTx patients who underwent ECP from those who did not. This component primarily comprised immune activation and inflammation-related genes (e.g. IL-1β, TNF-α, IL-17, MIP-1α, MIP-1β, MCP-1).

ECP downregulated IL-2 and IL-15 production, two essential cytokines for CTL and NK cell differentiation and maturation. This regulation is particularly significant, as it led to a dampening of immune cell-mediated effector mechanisms, evidenced by reduced CTL and NK cell activation in ECP patients. Notably, the percentage of degranulating, fully armed CTL and NK cells was significantly lower in ECP-treated patients, further confirming the therapy’s overall ability to suppress immune activation. ECP also resulted in the upregulation of anti-inflammatory immune cell populations and effector molecules: PD-1 and PDL1-expressing T helper lymphocytes as well as IL-10-producing Treg lymphocytes were significantly increased by ECP. PD-1 is a membrane protein that controls the magnitude of T-cell responses. Through the ligation of PD-L1, its main receptors, PD-1 modulates tolerance and inhibits T-cell mediated immunity, thus playing a fundamental role in immune responses; ECP induced an increase in lymphocytes expressing these proteins that are an important factor involved in the down-modulation of immune activation.

As would be expected in patients undergoing any kind of allograft transplantation, Th1 and TH17 lymphocytes were increased over time independently of ECP in both groups of LuTx individuals. The overall nature of immune response, though, stems from a complex and dynamic balance between activating and dampening signals and proteins. In patients receiving ECP the balance was clearly skewed toward a down-regulation of immune activation, the overall therapeutic goal in transplantation.

The ultimate proof that ECP-associated immune modulation leads to clinical benefit was the result of analysis of correlations between improvement in ppFEV_1_ and changes in immune parameters. Thus, an inverse correlation was detected between ppFEV_1_ and IL-8, IL-17 and IL-1β. IL-8 is a chemoattractant cytokine which distinct targets neutrophils, attracting and activating these cells in tissues. Neutrophils are usually the first leukocytes to infiltrate transplanted organs, where they organize the generation of extracellular trap formation to promote inflammation in the graft, thus being a well-established marker of transplant injury. IL-17 is a prototypical family of pro-inflammatory cytokines which are produced by TH-17 lymphocytes. IL-17 plays a pivotal role in maturation of DC progenitors, allogeneic T cell proliferation, and the expression of alloimmune reactivity, being a main actor in organ rejection. IFN-β, finally, is amongst the main proteins initiating the transcription of immune genes via activation of the JAK–STAT pathway, thus being essential in immune activation. The biological/clinical importance of these correlations is further underlined by the observation that ECP-induced improvement of ppFEV_1_ was positively correlated with BAL IL-1Ra concentration. IL-1Ra is a natural inhibitor of the pro-inflammatory effect of IL-1β, down-regulating the pleiotropic inflammatory responses initiated by this cytokine.

During the same years in which this pilot study was conducted, the Vienna group was carrying out a similar randomized study (12). However, the two studies differ in several aspects. The Viennese study enrolled patients with chronic obstructive pulmonary disease, followed a different post-transplant therapeutic regimen, and had a distinct ECP application protocol. Additionally, the Viennese study also used a composite endpoint, but it included high-grade AR, and CMV infection or the manifestation of CLAD within the first 24 months. The Viennese study, which had a larger sample size, demonstrated a significant reduction in both the number and severity of acute rejection, as well as a notable decrease in the cumulative incidence of CLAD. Additionally, the study reported a lower infection rate in the treatment arm, as we did.

The main limitation of our pilot study is the small sample size. Additionally, the COVID-19 pandemic outbreak hindered the enrollment of the target number of patients. We acknowledge that the sample size calculation was based on an expected reduction in acute rejection incidence and does not directly relate to the primary endpoints of safety and feasibility. Therefore, the sample size was not formally powered to assess these primary outcomes, which should be considered an additional limitation of the study. Nevertheless, as a pilot study, our research provides some indications: 1) ECP is well tolerated as induction therapy after lung transplantation, 2) a large multicenter randomized controlled trial could select an immunological parameter (CLT, NK, cytokines) as the main outcome and ppFEV_1_ as secondary outcome, 3) the immunomodulation caused by ECP promotes improvement in respiratory function that persists for many months after the end of therapy, 4) our advanced immunological research has shed new light on the mechanism of action of ECP opening new hopes for rebalancing immunosuppressive therapy in organ-transplanted patients.

Many questions remain open. The optimal timing, the minimal frequency and the duration of ECP as induction therapy remain to be elucidated, also the number of patients to be treated for a measurable and significant clinical outcome.

## Data Availability

The raw data supporting the conclusions of this article will be made available by the authors, without undue reservation.
