# Supplementary material for: Extracorporeal photopheresis as induction therapy in lung transplantation for cystic fibrosis: a pilot randomized trial
Source: Front Immunol. 2025 May 16;16:1583460. doi: 10.3389/fimmu.2025.1583460 (PMC12122324; doi:10.3389/fimmu.2025.1583460)
Supplement: Supplementary file 1 [file DataSheet1.docx]

Supplementary 1. Standard immunoppressive therapy

Glucocorticoids:

- Methylprednisolone 0.5 mg/Kg intravenously twice a day for the first three post-operative days
- Prednisone 0.5 mg/Kg orally starting from the fourth post-operative day.

Antimetabolites:

- Azathioprine 2 mg/Kg orally once a day
- Tacrolimus 0.2 mg/Kg orally once a day (trough level in serum: 10-15 ng/ml)

Anti-bacterial prophylaxis:

- Third-generation cephalosporin
- Co-trimoxazole 960 mg orally once a day, three days a week for 24 months

Anti-fungal prophylaxis: Voriconazole 200 mg orally (or via nasogastric tube) twice a day for 6 months

Anti-viral prophylaxis: Ganciclovir 5 mg/Kg IV twice a day for two weeks, then Valganciclovir 900 mg based on risk profile.

Supplementary 2. ECP treatment.

All extracorporeal photopheresis treatment were performed using a dedicated instrument (Therakos, CellexTM), the only FDA-approved device for “on-line” ECP, specifically bought for the project and used by experienced apheresis personnel. The dose of 8-MOP (UvadexTM) was calculated on the treatment volume collected during the plasma/buffy coat collection process using the following formula: treatment volume in ml x 0,017 = ml of 8-MOP required for the recirculation bag. The complete treatment was on-line without discontinuation of circulating blood.
